# Vaccination

**Published:** 1895-01

**Authors:** 


### HOMŒOPATHIC PHYSICIAN,

A MONTHLY JOURNAL OF

HOMŒOPATHIC MATERIA MEDICA AND CLINICAL MEDICINE.

"If our school ever gives up the strict inductive method of Hahnemann, we are lost, and deserve only to be mentioned as a caricature in the history of medicine."—CONSTANTINE HERING.

Vol. XV. JANUARY, 1895. No. 1.

### EDITORIAL.

VACCINATION.—The fear of a renewed invasion of small-pox during the present winter has once more aroused the frenzy for vaccination. Boards of Health are urging the people to be vaccinated, and they are enforcing it relentlessly in the public schools and among the police and firemen of the great cities. Especially is this true of Philadelphia. The city is divided into regular sections or districts, over each one of which is appointed a special vaccine physician, who makes the rounds of the schools in his own district vaccinating the pupils.

Of course, much damage is, in consequence, being done to children, and there seems to be no means of stopping the evil.

This journal has from time to time published authentic cases of death from vaccination. Many more must occur which are not heard of by the editor. In his own practice the editor has witnessed the death of a little girl eleven years old indirectly due to vaccination.

Another child was vaccinated by the regular vaccine physician, and the resulting ulceration was simply appalling. It had none of the characteristics of a vaccine pustule, yet it was pronounced by the vaccine doctor to have "taken" very well. It took months to cure this ulceration. Still another child has been afflicted with inflammation of the knee, with consequent deformity, brought about by vaccination.

Aside from any inherent evils of vaccination in itself, much of its danger arises from its glaring perversions by unscrupulous practitioners. Notwithstanding the disastrous results known to occur from arm-to-arm vaccination, there are many physicians who continue to practice it. Many still employ the scab, kept, possibly, for a couple of years before they have occasion to use it.

So-called vaccine virus is collected from cows that are not affected by the vaccine fever at all, but are under the influence of an inoculation with small-pox from the human subject. The resulting pustule, having a resemblance to the true vaccine, is assumed to be the true article by the speculative and arrogant intellects of the people who dare to engage in such infamous practice.

Under such circumstances all manner of filth is put into the circulation of the deluded victims who submit to the operation, and then the resulting ulceration is called vaccination without scruple.

It will thus be seen that the stipulation made by some anti-vaccinationists, that if vaccination must be performed only virus direct from the cow shall be used, falls short of accomplishing the good results intended.

If, as is so strenuously maintained, tuberculosis is highly contagious, why is not tuberculosis largely increased in the human being by his being vaccinated through infected virus taken from tuberculous cattle?

It would seem, from these and other considerations not touched upon in this article, that the whole practice of vaccination should be abolished.

Beneficial effects of vaccination are, of course, witnessed here and there, but the contradiction of testimony in these cases has been reconciled by the explanation given by Dr. Stuart Close in his able article entitled "Truth and Error in Vaccination," published in THE HOMŒOPATHIC PHYSICIAN for November, 1892, page 468.

The favorable results in a few cases where the vaccine was successful because, as Dr. Close shows, it was homœopathic to the case, secure for the practice a reputation which brings to it a host of victims just the same as a quack medicine gets an immense sale through the reputation of a few accidental cures.

Therefore the whole practice of vaccination should be abolished and the laws concerning it repealed.

